# Characterization of the complete mitochondrial genome of *Okenia hiroi* (Baba, 1938) (Nudibranchia, Goniodorididae)

**DOI:** 10.1080/23802359.2021.1901627

**Published:** 2021-03-19

**Authors:** Thinh Dinh Do, Dae-Wui Jung, Tae-June Choi, Hyung-Eun An, Chang-Bae Kim

**Affiliations:** a Department of Biotechnology, Sangmyung University, Seoul, Korea; b Korea Marine-Bio Laboratory, Daejeon, Korea

**Keywords:** *Okenia*, mitogenome, nucleotide composition, phylogeny

## Abstract

*Okenia* is a speciose genus of the family Goniodorididae with more than 50 valid species. The phylogenetic relationships within the genus are little known. The mitogenome is a good marker to understand the phylogenetic relationships of relative species. This study was performed to sequence the mitogenome of *O. hiroi*. The mitogenome of *O. hiroi* was 14,583 bp in size and was composed of 37 genes, including 13 protein-coding genes, two ribosomal *RNA* genes, and 22 *tRNA* genes. The nucleotide composition was 30.5% A, 13.6% C, 16.5% G, and 39.4% T. The phylogenetic analysis showed that *O. hiroi* is sister to *Notodoris gardineri* (Aegiridae). This study recorded the first mitochondrial genome sequence of the family Goniodorididae.

The genus *Okenia* is composed of small to medium-sized nudibranchs (Pola et al. 2019). Because of its diversity, many new species of the genus *Okenia* have been recently described (Gosliner 2004; Pola et al. 2019). Currently, the genus includes more than 50 valid species that are distributed from the Pacific Ocean to the north and south Atlantic Ocean (Pola et al. 2019; Horton et al. 2021). In addition, there are limited studies on the phylogeny of the genus (Pola et al. 2019). *Okenia hiroi* is a species of the genus *Okenia*. This species was described from Japan and reported from Korea and Hong Kong (Jung et al. 2014). The analyses of the mitogenome genes of *O. hiroi* might be useful for understanding molecular identification and phylogeny of the genus. Therefore, this study aims at sequencing and analyzing structure of the mitogenome of *O. hiroi.*

The specimen of *O. hiroi* was collected from Munamjin-ri, Korea (38°18′14.75′′N, 128°34′1.05) in June 2020. Following the collection, the specimen (voucher number: SMU00087) was preserved in absolute ethanol and deposited at the Department of Biotechnology, Sangmyung University, Korea. Total DNA was extracted from the foot of the specimen. The extracted DNA was checked for puriyand concentration using a MaestroNano spectrophotometer (Maestrogen, Hsinchu, Taiwan). After library preparation, mitogenome sequencing was performed with MGISEQ-2000 (MGI, Shenzhen, China). MITObim (Hahn et al. 2013) was used for sequence assembly and MITOS (Bernt et al. 2013) was used for sequence annotation. Mitogenome sequences representing different families of the suborder Doridina and two species of the suborder Cladobranchia available in GenBank were obtained for phylogenetic analysis. All 37 genes of the mitogenomes were used for tree reconstruction. The partition schemes and best-fit model were searched in PartitionFinder 2 (Lanfear et al. 2017). MrBayes version 3.2.7a was applied to investigate the phylogenetic position of *O. hiroi* (Ronquist et al. 2012). For analysis, 10,000,000 generations were run and sampling was conducted every 1,000 generations.

The mitogenome of *O. hiroi* (GeneBank accession number: MW408699) was 14,583 bp long and contained 13 protein-coding genes, two *rRNA* genes, 22 *tRNA* genes. The nucleotide composition of the mitogenome was 30.5% A, 13.6% C, 16.5% G, and 39.4% T, with an A + T content of 69.9%. The size of 13 PCGs ranged from 156 bp (*atp8*) to 1,623 bp (*nd5*). Of 13 PCGs, there were nine genes (*cox1*, *cox2*, *cytb*, *nad1*, *nd2*, *nad4*, *nad4l*, *nd5*, and *nd6*) encoded on the H-strand and four remaining genes (*atp6*, *atp8*, *cox3*, and *nd3*) encoded on the L-strand. The mitogenome contained two *rRNA* genes with the size of 747 bp and 1,103 for 12S rRNA and 16S rRNA, respectively. Of the 22 tRNA genes, tRNA-Ser was the shortest gene (58 bp) and tRNA-Asn and tRNA-Asp were the longest genes (69 bp).

There were 27 intergenic regions in the mitogenome of *O. hiroi*, ranging from 1 to 108 bp. The longest intergenic region (108 bp) was located between cox1 and tRNA-Val genes. Compared to the other available mitogenomes of nudibranchs, this is the longest intergenic region between *cox1* and tRNA-Val genes so far recorded. Also, there were five overlapping regions in the mitogenome of *O. hiroi*. The longest overlapping region (20 bp) was located over *nd1* and *nd5* genes.

Figure 1 shows the phylogenetic position of *O. hiroi* in the suborder Doridina. As seen from Figure 1, all species of Doridina were clustered together. The phylogenetic tree indicated that *O. hiroi* formed a clade with *Notodoris gardineri* (Aegiridae). In term of systematics, both Goniodorididae and Aegiridae belong to the super family Onchidoridoidea (Bouchet et al. 2017). Previous study also revealed a close relationship between Goniodorididae and Aegiridae (Hallas et al. 2017). In the future study, additional mitogenomes of the family Goniodorididae should be generated to investigate the relationships within the family.

**Figure 1.**
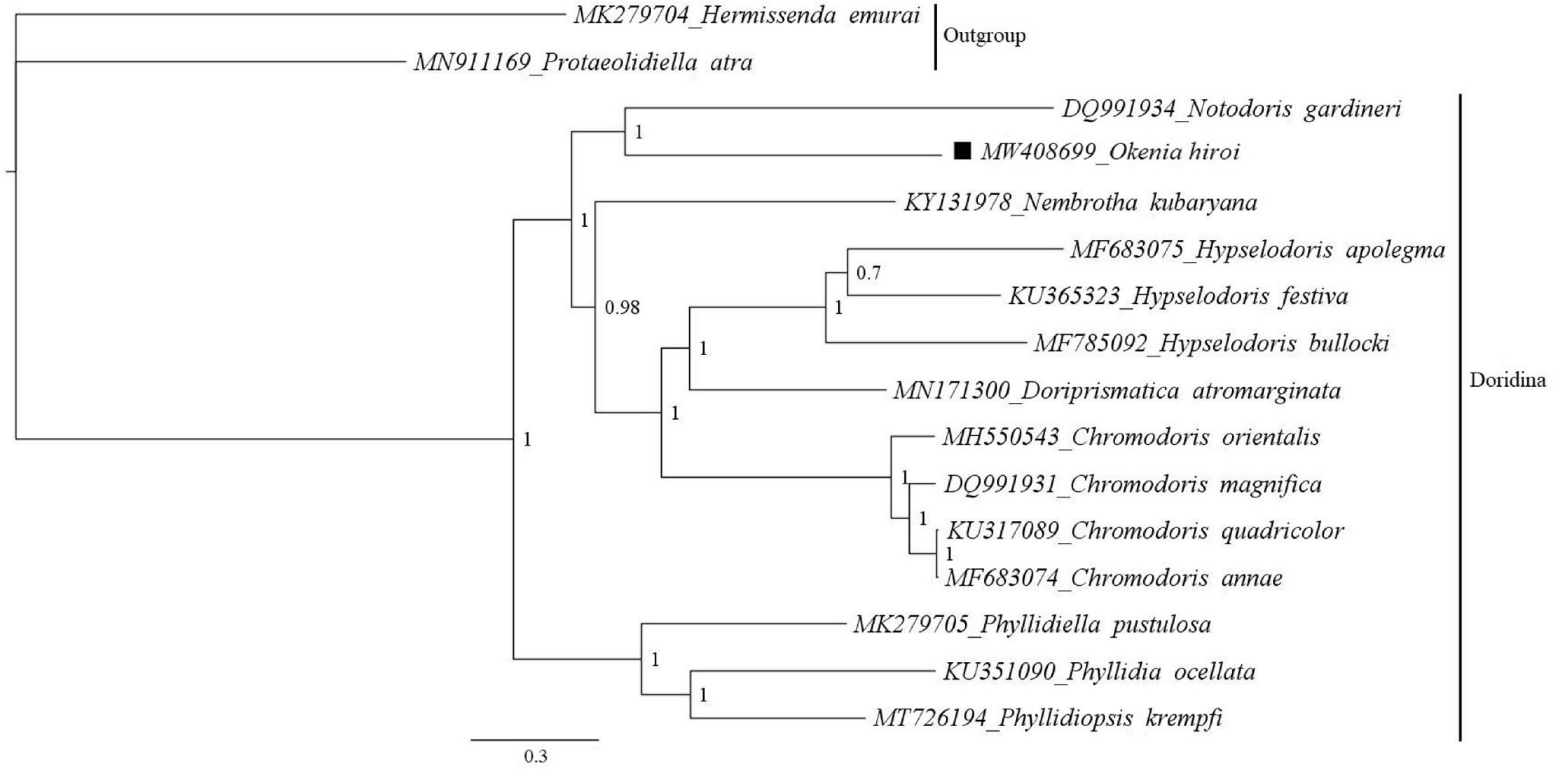
Phylogenetic position of *Okenia hiroi* in the suborder Doridina. GenBank accession numbers are put next to the species names. Two species from the suborder Cladobranchia (*Hermissenda emurai* and *Protaeolidiella atra*) are used as the outgroup. Posterior probability values are shown at the nodes.

## Data Availability

The genome sequence data that support the findings of this study is openly available in GenBank of NCBI at (https://www.ncbi.nlm.nih.gov/) under the accession no. MW408699. The associated BioProject, SRA, and Bio-Sample numbers are PRJNA693411, SRR13555242, and SAMN17386532, respectively.

## References

[CIT0002] Bernt M, Donath A, Jühling F, Externbrink F, Florentz C, Fritzsch G, Pütz J, Middendorf M, Stadler PF. 2013. MITOS: improved de novo metazoan mitochondrial genome annotation. Mol Phylogenet Evol. 69(2):313–319. 22982435 10.1016/j.ympev.2012.08.023

[CIT0003] Bouchet P, Rocroi JP, Hausdorf B, Kaim A, Kano Y, Nützel A, Parkhaev P, Schrödl M, Strong EE. 2017. Revised classification, nomenclator and typification of gastropod and monoplacophoran families. Malacologia. 61(1–2):1–526.

[CIT0004] Gosliner TM. 2004. Phylogenetic systematics of *Okenia*, *Sakishimaia*, *Hopkinsiella* and *Hopkinsia* (Nudibranchia: Goniodorididae) with descriptions of new species from the tropical Indo-Pacific. Proc Calif Acad Sci. 5:125–161.

[CIT0005] Hahn C, Bachmann L, Chevreux B. 2013. Reconstructing mitochondrial genomes directly from genomic next-generation sequencing reads-a baiting and iterative mapping approach. Nucleic Acids Res. 41(13):e129–e129. 23661685 10.1093/nar/gkt371 PMC3711436

[CIT0006] Hallas JM, Chichvarkhin A, Gosliner TM. 2017. Aligning evidence: concerns regarding multiple sequence alignments in estimating the phylogeny of the Nudibranchia suborder Doridina. R Soc Open Sci. 4(10):171095. 29134101 10.1098/rsos.171095 PMC5666284

[CIT0007] Horton T, Kroh A, Ahyong S, Bailly N, Boyko CB, Brandão SN, Gofas S, et al. 2021. World register of marine species. VLIZ. [accessed 02 Feb 2021]. https://www.marinespecies.org.

[CIT0008] Jung D, Lee J, Kim CB. 2014. A report on five new records of nudibranch molluscs from Korea. Anim Syst Evol Divers. 30(2):124–131.

[CIT0009] Lanfear R, Frandsen PB, Wright AM, Senfeld T, Calcott B. 2017. Partition finder 2: new methods for selecting partitioned models of evolution for molecular and morphological phylogenetic analyses. Mol Biol Evol. 34(3):772–773. 28013191 10.1093/molbev/msw260

[CIT0010] Pola M, Paz-Sedano S, Macali A, Minchin D, Marchini A, Vitale F, Licchelli C, Crocetta F. 2019. What is really out there? Review of the genus *Okenia* Menke, 1830 (Nudibranchia: Goniodorididae) in the Mediterranean Sea with description of two new species. PLoS One. 14(5):e0215037. 31042722 10.1371/journal.pone.0215037 PMC6493716

[CIT0011] Ronquist F, Teslenko M, Mark PVD, Ayres DL, Darling A, Hohna S, Larget B, Liu L, Suchard MA, Huelsenbeck JP. 2012. MrBayes 3.2: efficient Bayesian phylogenetic inference and model choice across a large model space. Syst Biol. 61(3):539–542. 22357727 10.1093/sysbio/sys029 PMC3329765

